# DNA degradation in fish: Practical solutions and guidelines to improve DNA preservation for genomic research

**DOI:** 10.1002/ece3.6558

**Published:** 2020-07-13

**Authors:** Tom Oosting, Elena Hilario, Maren Wellenreuther, Peter A. Ritchie

**Affiliations:** ^1^ School of Biological Sciences Victoria University of Wellington Wellington New Zealand; ^2^ The New Zealand Institute for Plant & Food Research Ltd Auckland New Zealand; ^3^ Nelson Seafood Research Unit The New Zealand Institute for Plant & Food Research Ltd Nelson New Zealand; ^4^ School of Biological Sciences The University of Auckland Auckland New Zealand

**Keywords:** DMSO, DNA preservation, ethanol, fish, next‐generation sequencing, next‐generation sequencing, snapper

## Abstract

The more demanding requirements of DNA preservation for genomic research can be difficult to meet when field conditions limit the methodological approaches that can be used or cause samples to be stored in suboptimal conditions. Such limitations may increase rates of DNA degradation, potentially rendering samples unusable for applications such as genome‐wide sequencing. Nonetheless, little is known about the impact of suboptimal sampling conditions. We evaluated the performance of two widely used preservation solutions (1. DESS: 20% DMSO, 0.25 M EDTA, NaCl saturated solution, and 2. Ethanol >99.5%) under a range of storage conditions over a three‐month period (sampling at 1 day, 1 week, 2 weeks, 1 month, and 3 months) to provide practical guidelines for DNA preservation. DNA degradation was quantified as the reduction in average DNA fragment size over time (DNA fragmentation) because the size distribution of DNA segments plays a key role in generating genomic datasets. Tissues were collected from a marine teleost species, the Australasian snapper, *Chrysophrys auratus*. We found that the storage solution has a strong effect on DNA preservation. In DESS, DNA was only moderately degraded after three months of storage while DNA stored in ethanol showed high levels of DNA degradation already within 24 hr, making samples unsuitable for next‐generation sequencing. Here, we conclude that DESS was the most promising solution when storing samples for genomic applications. We recognize that the best preservation protocol is highly dependent on the organism, tissue type, and study design. We highly recommend performing similar experiments before beginning a study. This study highlights the importance of testing sample preservation protocols and provides both practical and economical advice to improve DNA preservation when sampling for genome‐wide applications.

## Graphical abstract

The initial stages of DNA preservation can be crucial for obtaining high‐quality data for genomic research. However, environmental conditions in the field and during transport are typically suboptimal for preserving DNA, potentially spoiling costly samples. Here, we evaluate two cost‐effective preservation solutions and provide a comprehensive background on what causes DNA to degrade and practical guidelines on how to ensure the best approach for preserving DNA in field‐collected samples.

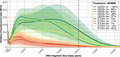

## 1 INTRODUCTION

Next‐generation sequencing (NGS) applications are now widely applied to population genomic studies of nonmodel species, addressing questions related to the conservation, ecology, evolution, and demography of species (Ellegren, 2014). These applications typically require high‐molecular‐weight (HMW) DNA (>20 kbp) for library preparation and sequencing to obtain high‐quality genomic datasets. Such requirements are more demanding than those for traditional PCR‐based approaches where a wide range of DNA qualities can be used, as targeted DNA sequences are seldom longer than 5 kbp. As a consequence, earlier methods for preserving samples for genetic analysis may perform suboptimally and fail to meet requirements of DNA preservation for genomic research.

DNA is ideally extracted immediately after tissue sampling or stored at subzero temperatures and extracted shortly after (e.g., within 24 hr). However, field conditions and limited funding often restrict the resources available for sample preservation. This forces researchers to work within tight boundaries to prevent DNA degradation as much as possible. The level of preservation required to obtain high‐quality DNA will vary depending on the goals of the study and environmental conditions, time spent in the field, available resources, and type of tissue collected. The solutions in which tissues are preserved provide another layer of added flexibility. Two of the most common and also cost‐effective solutions to preserve tissue for DNA extraction are ethanol and DESS (20% DMSO, 0.25 M EDTA, NaCl saturated solution; Yoder et al., 2006). However, it is less clear how suitable these solutions are for genomic research when preservation conditions are variable and when the period over which tissues are stored spans multiple months.

Previous studies that assessed sample preservation using various storage solutions had quantified DNA quality based on the quantity extracted or ability to sequence short DNA fragments (<5 kbp; Asahida, Kobayashi, Saitoh, & Nakayama, 1996; Bainard, Klironomos, & Hart, 2010; Dawson, Raskoff, & Jacobs, 1998; Gorokhova, 2005; Graham et al., 2015; Graham, Turk, & Rutty, 2008; Michaud & Foran, 2011; Seutin, White, & Boag, 1991; Stein, White, Mazor, Miller, & Pilgrim, 2013). Such metrics do not provide an accurate assessment of DNA quality for most genomic sequencing applications, as they do not provide any information regarding integrity of the DNA on large scales. Among the studies mentioned above, time periods over which sample preservation was assessed varied greatly, spanning from 12 hr up to three years (Graham et al., 2008, 2015). When included in the study design, DESS solution was found to preserve DNA best according to the quality metrics of the study in question (Dawson et al., 1998; Michaud & Foran, 2011; Seutin et al., 1991). We chose DNA fragment size as a proxy for DNA quality because it is a key metric used by sequencing providers for generating representative genomic datasets with practically any sequencing platform. Here, we will refer to DNA degradation as double‐stranded breaks resulting in a reduction in average fragment size. DNA changes that do not result in a reduction in fragment size will be referred to as DNA damage.

### 1.1 Why is high‐molecular‐weight DNA important?

The effect of DNA quality (i.e., fragment size) on genomic datasets can vary depending on how the data is generated. Three of the most common sequencing applications in genomic research are RADseq, whole‐genome sequencing, and long‐range sequencing. RADseq approaches include popular methods such as genotype‐by‐sequencing (GBS), restriction‐site associated DNA (RAD) sequencing, and double‐digest restriction‐site‐associated DNA (ddRAD) sequencing (Andrews, Good, Miller, Luikart, & Hohenlohe, 2016). With RADseq, small sections of the genome which are consistently sequenced across samples. RADseq is more cost‐effective and particularly popular when dealing with nonmodel organisms, and organisms that have large genome sizes such as many plants species (Clugston et al., 2019). RADseq protocols use one (RAD) or two restriction enzymes (ddRAD) in order to cut genomes of individuals at common sites and subsequently isolate a set of fragments, usually between 300 and 500 bp long. Based on the restriction enzyme cut sites, specific regions of the genome can be consistently targeted for sequencing in all individuals. DNA degradation affects the efficiency of reduced presentation sequencing, increasing the number of missing loci. For instance, Guo, Yang, Chen, Li, and Guo (2018) showed that degraded samples resulted in the reduction of total reads, and a reduction in the number of reads that successfully mapped to the reference genome using ddRAD. Similar results were observed by Graham et al. (2015), where incubation at room temperature of samples up to 96 hr induced DNA degradation reduced the final numbers of SNPs. These studies showed that RADseq performs best when using high‐molecular‐weight DNA to generate population data.

Whole‐genome sequencing (generally referred to as WGS), or whole‐genome resequencing involves sequencing of the entire genome for one or multiple organisms. Whole‐genome resequencing allows for some variation in DNA quality because it does not rely on restriction enzymes for fragmentation. Instead, during library preparation DNA is fragmented into small sizes (e.g., 300–500 bp) using sonication or a mechanical method. However, high‐quality DNA provides a more balanced read distribution across the genome per sample, and a consistent coverage across samples (Anderson et al., 2010). With long‐range sequencing applications such as PACBio and Oxford Nanopore technologies, the effect of DNA degradation is a reduction in read length. PACBio and MinION are able to sequence fragments >80 kbp up to 100 kbp (van Dijk, Jaszczyszyn, Naquin, & Thermes, 2018), and the level of DNA degradation will drastically influence the average fragment size that can be obtained. We recommend using DNA obtained from fresh or flash‐frozen samples for long‐range sequencing. Moreover, for genome assemblies, DNA quality should be sufficiently high to provide large number of reads evenly distributed along the genome, creating long, continuous scaffolds for a genome assembly (Dominguez Del Angel, 2018). We recommend using DNA obtained from fresh samples for genome assemblies.

### 1.2 What influences the rate of DNA degradation?

DNA degradation and DNA damage occur through enzymatic processes, oxidative damage, UV radiation, and hydrolysis (Schroeder, Lad, Wyman, Williams, & Wolfenden, 2006). DNA degradation starts within minutes or hours after sampling from a live specimen (Graham et al., 2015) and will continue to degrade regardless of how the DNA has been preserved (Guo et al., 2018). Endonucleases and exonucleases can lead to the rapid break down of DNA inside the cells. As any enzyme activity is sensitive to temperature, the degradation process is reduced at lower temperatures. Thus, keeping samples cold will slow down the enzymatic degradation of DNA. In addition, oxidative damage by free radicals and hydrolysis through interaction with water (especially acidic water) compromises DNA integrity. Tissue samples will always be, to some extent, subject to all the processes presented above during transport. Finally, UV radiation from direct sunlight can induce double‐stranded DNA damage and form T‐T dimers.

Once extracted, DNA continues to degrade even while being stored under optimal conditions (i.e., low temperature, buffered media, sterile environment, and/or minimal manipulations; Guo et al., 2018). Storing extracted DNA in a solution that buffers the pH (e.g., Tris‐HCl pH 8) protects samples from oxidative damage and hydrolysis of phosphate bonds, increasing the chance of retaining good DNA quality. Tris‐HCl buffering is often combined with ethylenediaminetetraacetic acid (EDTA), commonly known as TE buffer. EDTA binds to metal ions, deactivating metal‐dependent enzymes such as DNase. It is worth noting that high concentrations of EDTA can also inhibit enzymatic activity during library preparation and should be reduced as much as possible prior to library preparation (preferably to <0.1 mM; Sambrook, Fritsch, & Maniatis, 1989).

### 1.3 Experimental setup

We evaluated the effect of two widely applied storage solutions (DESS and ethanol) and preservation treatments (heat treatment and storage temperature) on DNA degradation over time (1 day, 1 week, 2 weeks, 1 month, and 3 months). Samples were stored under suboptimal storage conditions for 3 months (ambient room temperature of around 20°C and in a cold room set to 5°C). Our goal was to evaluate how different preservation methods effect the initial stages of sample preservation and choose the right protocol for our study. We extracted DNA from the marine teleost species the Australasian snapper *Chrysophrys auratus*, which has been observed to experience high rates of DNA degradation within the first 24 hr after sampling (M. Wellenreuther, personal observation). Controlling the initial stage of DNA sample preservation was considered crucial to this study for obtaining good quality genomic data from natural snapper populations.

## 2 METHODS

**FIGURE 1:**
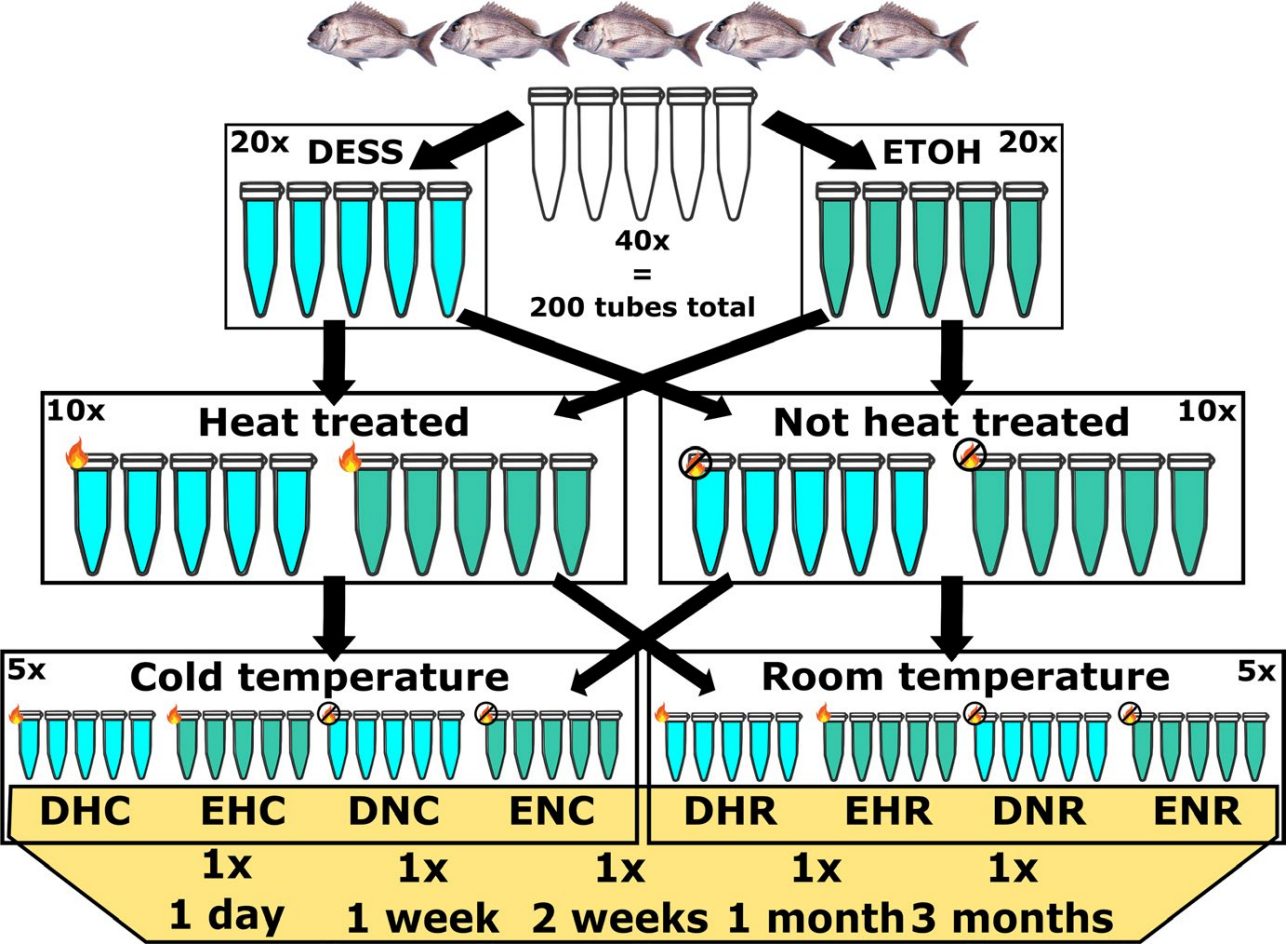
Sampling and experimental setup. In total, 200 fin clips were collected from 5 fish (40 per individual). Samples were exposed to 3 storage treatments (solution, heat treatment, storage temperature) and time. DESS: solution of 20% DMSO, 0.25 M EDTA, and NaCl saturated. ETOH: 99.5% ethanol. Heat‐treated samples were kept at 80°C for 5 min within 1 hr of sampling. Samples kept at “room” and “cold” temperatures were stored at 20°C and 5°C, respectively. All samples were stored under dry dark conditions

Fin clips (approximately 1 cm^2^) were collected from the anal and caudal fin (avoiding the cartilage tissue as much as possible) from five Australasian snapper (all of the same age and kept under the same environmental conditions) from the Seafood Research Unit in Nelson operated by The New Zealand Institute of Plant and Food Research Limited. We used three storage treatments: a preservation solution, a heat treatment, and a storage temperature (Figure 1). In total, 200 fin clips were individually stored in 2.0‐ml sterile O‐ring tubes, with 1.5 ml preservation solution. This way, each sample was fully submerged in the solution and not in contact with other samples, which could potentially inhibit the solution from entering the tissue. Samples were collected within a two‐hour time window, cleaning sampling gear with ethanol between individuals. The fin clips were extracted at five different time points: 1 day, 1 week, 2 weeks, 1 month, and 3 months. The two preservation solutions used in this study were ethanol (absolute > 99.5%, grade: molecular biology) and DESS (20% DMSO, 0.25 M EDTA, sodium chloride saturated solution, see Appendices S1 and S2). Ethanol dehydrates cells and causes proteins to coagulate in order to preserve the sample by inhibiting cellular processes that may breakdown the DNA. DESS prevents DNA degradation by deactivating metal‐dependent enzymes (i.e., DNase) using EDTA. In addition, our EDTA 0.5 M stock solution was set at pH 8 with NaOH to dissolve the EDTA, creating a pH buffering effect. The solution was salt statured with sodium chloride (NaCl) which stabilizes the DNA (MacFarlane et al., 2017). Finally, DMSO is known to transport compounds (e.g., EDTA and NaCl) across cell membranes and serves as a cryogenic (Seutin et al., 1991). DESS is known to perform well under a wide range of temperatures, including room temperature. Heat‐treated samples were incubated at 80**°**C for 5 min within an hour of sampling to destroy DNA degrading enzymes. Samples were stored at a “cold” temperature of 5°C and at room temperature (~20°C). Temperatures were chosen to resemble the storage conditions typically encountered in the field and generally considered suboptimal for storage over multiple days. All samples were stored under dark conditions. Five technical replicates from each individual were extracted for each combination of preservation treatments over five different time intervals (Figure 1), creating a total of 200 DNA extractions.

DNA was extracted using a high‐salt extraction protocol adapted from Aljanabi and Martinez (1997) (see Appendices S1 and S2). To standardize the amount of DNA extracted from each sample, between 30 and 40 mg of tissue was used for each extraction. All extractions were performed by the same operator. Wide‐bore pipette tips were used every time DNA was pipetted from between tubes to reduce physical stress on the DNA fragments. DNA was eluted overnight at room temperature in 100 µl TE buffer (10 mM Tris‐HCl pH 8, 1 mM EDTA). Impurities and DNA concentration were measured using an Implen^©^ NP80 spectrophotometer and Qubit broad range kit (Invitrogen^©^). Samples were diluted to a concentration of ~3.0 ng/µl and assessed on the Fragment Analyzer (Advanced Analytical) using the high‐sensitivity genomic DNA analysis kit, and PROsize V3.0.1.6 to summarize the results.

A multi‐factor ANOVA was performed to test which variables had a significant effect on preservation condition of the sample and DNA fragmentation. DNA fragmentation was quantified using GQN quality score implemented in PROsize, based on the running front against known fragment sizes in the HS genomic DNA ladder. We performed three separate multi‐factor ANOVAs: One using all samples and two using samples from each of the solution treatments. Visualization of fragment size decay per treatment over time was done by assessing the electropherograms (implemented in PROsize). Electropherograms were merged using a custom Python script. Slight differences between each run resulted in different point estimates for larger fragment sizes. To create overlapping data points between each of the three fragment analyzer runs, fragment sizes above 1 kbp were rounded up to the nearest 10, and relative fluorescence unit (RFU) values were averaged across matching fragment sizes. Similarly, fragment sizes above 10 kbp were rounded to the nearest 100 and again RFU values were averaged. Negative RFU values were clipped to zero. Mean RFU values per treatment were calculated after subsetting, and 95% confidence intervals were estimated and plotted in R (R Core Team, 2013).

Finally, the area under each curve was estimated using DescTools (Signorell, 2016). We estimated the area under each curve per treatment to assess the relative abundance of HMW DNA in total amount of DNA. Fragment sizes smaller than 250 bp were not taken into account to prevent biases from RNA. The standardized percentages of high‐molecular‐weight were obtained by taking the plot with largest contribution of high‐molecular‐weight (i.e., largest area under the curve above 20 kbp) and using that to standardize the high‐molecular‐weight contributions from other treatments.

## 3 RESULTS

Our results show that the preservation treatments have a clear effect on the rate of DNA degradation. The multi‐factor ANOVA showed that the variable *solution* had a significant effect on DNA degradation, *p*‐value: 5.07 × 10^–12^ (Table 1). In addition, interactions between variables were mildly significant, suggesting interactions between *temp:time* and *solution:temp:time*, *p*‐values: .020 and .033, respectively. However, no significant difference was found between the ANOVA including all treatments, and the AVOVA only including *solution* as a variable, *p*‐value: .311. Patterns changed when samples from each *solution* were analyzed independently (Table 1). Samples stored in DESS showed a significant interaction between *temp:time*, *p*‐value: .020. This was also observed when all samples were combined. DNA fragmentation for samples stored in ethanol was significantly influenced by the heat treatment, *p*‐value: .007. Finally, an interaction between *heat:temp* was observed for samples stored in ethanol, *p*‐value: .036.

**TABLE 1:** Multi‐factor ANOVA

Treatment	*p*‐Value		
	All	DESS	ETOH
Solution	5.07e−12***		
Heat	.657	.803	.007**
Temp	.140	.228	.223
Time	.714	.419	.155
Solution: Heat	.354		
Solution: Temp	.384		
Heat: Temp	.372	.145	.036*
Solution: Time	.195		
Heat: Time	.979	1.000	.457
Temp: Time	.020*	.023*	.828
Solution: Heat: Temp	.052		
Solution: Heat: Time	.998		
Solution: Temp: Time	.033*		
Heat: Temp: Time	.738	.928	.338
Solution: Heat: Temp: Time	.995		

Note

*p*‐Values below .05, .01, and .001 are indicated by *, **, and ***, respectively. Solution: solution used to store samples, Heat: heat treatment, temp: storage temperature, time: period samples were stored until extraction.

**FIGURE 2:**
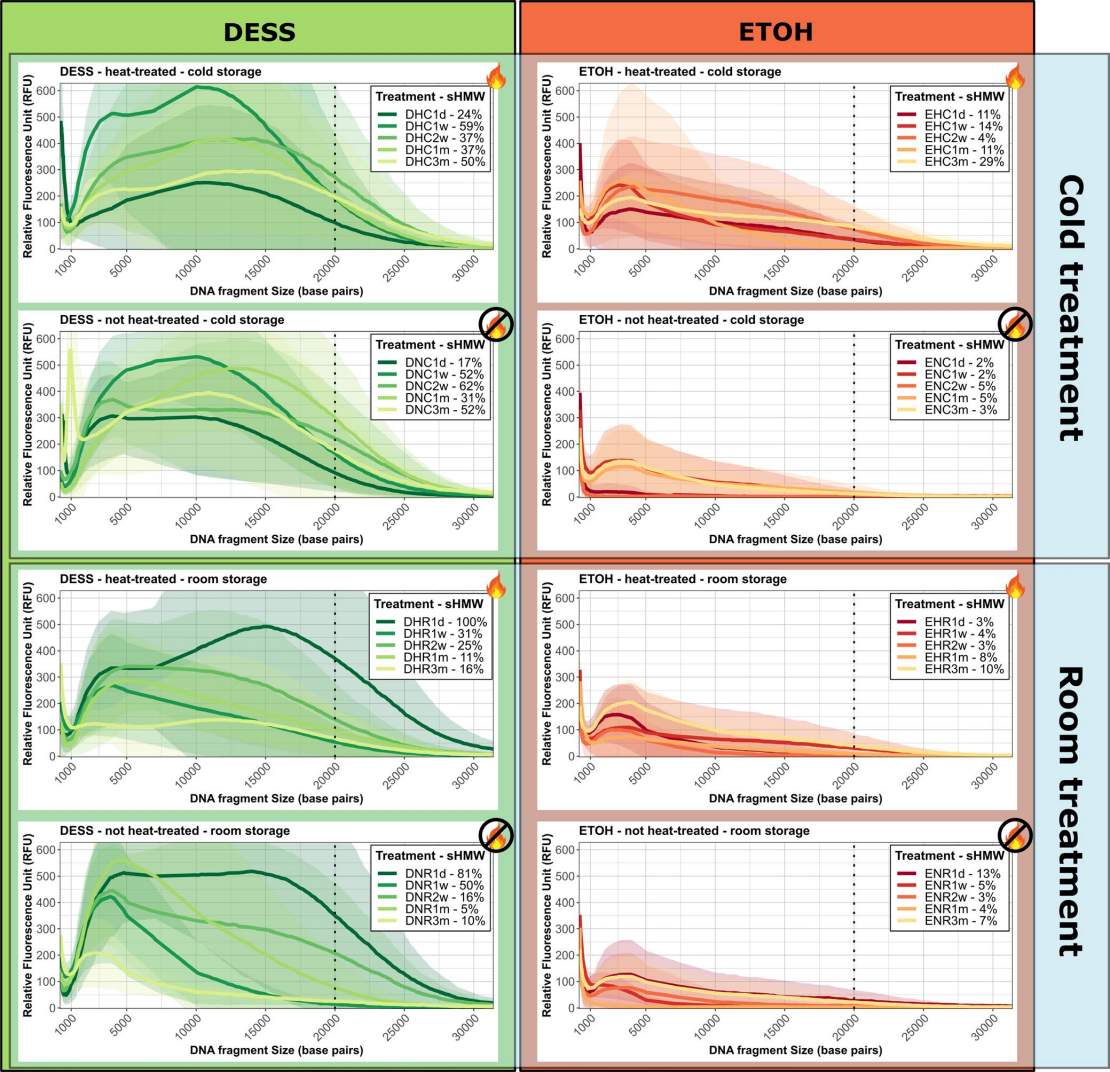
Electropherograms of DNA fragment over time for each treatment combination. High‐molecular‐weight (HMW) DNA is indicated at the right of the dotted line. Lines indicated mean RFU values over all samples stored in the respective treatment, shaded areas indicate the 95% confidence intervals, and the dotted line indicates 20 kbp intersect (high‐molecular‐weight DNA boundary). Each plot shows degradation over time: 1 day (1 d), 1 week (w), 2 weeks (2 w), 1 month (1 m), and 3 months (3 m). sHMW% indicates standardized high‐molecular‐weight DNA content for each treatment and time (percentages are standardized to the sample with the highest contribution of HMW, i.e., DNR1d). The flame icon indicates samples that have been heat treated

DNA fragmentation profiles over time show a clear effect of treatments on DNA degradation (Figure 2). Samples stored in DESS showed better preservation over time compared to samples stored in ethanol. However, sample preservation using DESS was highly variable, sometimes not following a linear degradation over time. DNA stored in ethanol was highly degraded after one day. However, heat treating and storing the samples at a lower temperature had an observable effect. Standardized percentages of high‐molecular‐weight DNA content showed that sampled stored in DESS and kept at 5**°**C still had >50% of high‐molecular‐weight DNA after 3 months, compared to the highest observed in the experiments. This shows preservation was relatively stable in those treatments over of the duration of the experiment.

**FIGURE 3:**
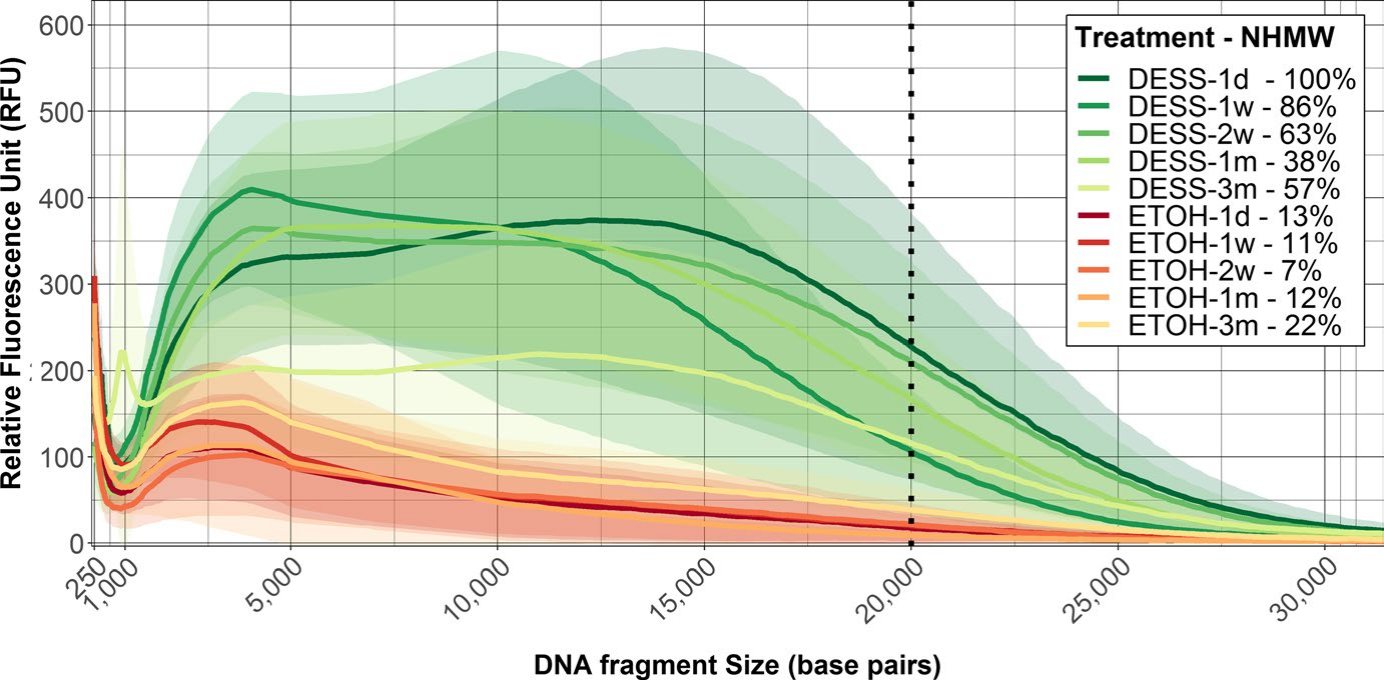
Electropherogram of DNA fragment size per solution treatment over time. *N* = 20 for each treatment. High‐molecular‐weight (HMW) DNA is indicated at the right of the dotted line, ETOH: ethanol 99.5%, DESS: DMSO, EDTA, and salt‐saturated solution. Lines indicated mean RFU values over all samples stored in the respective solution, shaded areas indicate the 95% confidence intervals, and the dotted line indicates 20 kbp intersect (high‐molecular‐weight DNA boundary). Each plot shows degradation over time: 1 day (1 d), 1 week (w), 2 weeks (2 w), 1 month (1 m), and 3 months (3 m). sHMW% indicates standardized high‐molecular‐weight DNA content for each treatment and time (percentages are standardized to the sample with the highest contribution of HMW, i.e., DESS‐1d)

The effect of the solution treatment is clearly observed when samples were pooled per solution treatment (Figure 3). However, DNA from samples stored in ethanol showed significant signs of degradation after one day. In contrast, DNA from samples stored in DESS appeared to maintain stable levels of HMW DNA fragments for up to one month following sample collection. Evidence of DNA degradation was detected in the DESS treatment after 3 months, observed by an increase of fragment sizes around 1 kbp and the frequency reduction of >20 kbp fragments.

The proportion of HMW DNA for all treatment groups was <16% (Table 2). As a proxy, samples stored for 24 hr in DESS were used as a standardized baseline because DNA was best preserved in that group. Over the first 2 weeks, samples stored in DESS had over 7.5 times more HMW DNA compared to the ethanol treatment (Table 2). After 3 months, samples stored in DESS still had twice the amount of HMW DNA compared to samples stored in ethanol.

**TABLE 2:** Proportion of high‐molecular‐weight DNA (20 kbp) over time when samples are pooled per solution treatment

Treatment	High‐molecular‐weight DNA %		High‐molecular‐weight DNA % (standardized)		Scale factor between DESS and ETOH
	DESS	ETOH	DESS	ETOH	DESS/ETOH
1 day	15.8	11.4	100.0	13.3	7.5
1 week	14.1	9.6	85.6	10.8	7.9
2 weeks	11.1	6.6	62.2	6.9	9.0
1 month	7.0	9.1	37.9	12.2	3.1
3 months	15.1	11.9	57.3	21.9	2.6

Note

DESS: solution of 20% DMSO, 0.25 M EDTA, and NaCl saturated. ETOH: 99.5% ethanol. The standardized percentages of high‐molecular‐weight were obtained by taking the plot with largest contribution of high‐molecular‐weight (i.e., largest area under the curve above 20 kbp, DESS—1 day) and using that to standardize all other high‐molecular‐weight contributions.

## 4 DISCUSSION

This study showed that sample preservation significantly influences the proportion and quality of extracted HWM DNA from fish samples. DESS was better at preserving DNA than ethanol under the conditions in which they were tested. Based on these findings, we conclude that DESS is best suited for preserving DNA for NGS applications. Our observations were consistent with previous studies that had included DESS in their experimental design (Dawson et al., 1998; Gaither, Szabo, Crepeau, Bird, & Toonen, 2011; Michaud & Foran, 2011; Seutin et al., 1991). Although these studies used different metrics to quantify DNA quality, DESS was consistently found to outperform ethanol under a range conditions. Regardless, ethanol will likely remain a popular choice for sample preservation. However, we recommend testing how well various preservation solutions perform based on the environmental conditions experienced in the field. Sample collection is the first step in any research project, which often involves a tremendous amount of effort from many people. Based on results of this study, we argue that it is worth evaluating the method of preservation to ensure samples will be yield the best data possible.

The multifactorial ANOVA suggested that solution type was the main factor contributing to the variation observed across DNA samples (Table 1). Storage temperature and time only had a significant effect when DESS was included in the analyses. This finding supports the notion that samples stored at lower temperatures degrade at lower rates, and that the rate is dependent on the storage solution (Dawson et al., 1998; Tsuji, Ushio, Sakurai, Minamoto, & Yamanaka, 2017). This is confirmed by the significant interaction between the variables *solution:temp:time*. Initial levels of DNA degradation for samples stored in ethanol were likely too high for any significant temporal patterns to be observed. DNA degradation in samples stored in ethanol was significantly influenced by heat treatment (Table 1). This suggests that heat‐treating samples for five minutes at 80°C are able to denature DNA degrading enzymes. It is unknown if heat‐treating samples for more than five minutes further inhibits enzyme‐mediated DNA degradation. Regardless, DESS appeared to be far more effective at inactivating such enzymes. Finally, the interaction between *heat:temp* for samples stored in ethanol suggests that storage solution only affects DNA degradation when samples are heat treated. Again, this is likely caused by high initial levels of DNA degradation, masking the effect of storage temperature.

The better performance of DESS compared to ethanol can be observed in the fragment size distribution over time (Figure 3). DNA stored in ethanol was significantly more degraded after one day, while DNA stored in DESS appeared relatively stable over the first month. The drastic reduction in HWM DNA in ethanol after one day suggests enzymes were actively degrading the DNA. It is possible that the high concentration of ethanol has caused proteins close to the cell wall to coagulate too fast, creating a protein barrier that prevents the ethanol from reaching further into the cell. Consequently, enzymes continue to function, resulting in DNA degradation. Lower concentrations of ethanol (e.g., 70%) would allow the ethanol to reach further into the cell, and however, this also reduces the effectiveness of the solution. Michaud and Foran (2011) tested three different concentrations of ethanol (i.e., 40, 70, and 100), but still found DESS to be most effective for preserving DNA. Further, the wide 95% confidence intervals do indicate a noticeable variation among individual samples. The observed variation is likely caused by the nested treatments within the experiment. Samples stored in DESS did show clear degradation after 3 months of storage. It is unclear what exact processes contributed to the observed degradation. It is possible that enzymes slowly start degrading the DNA over time, or chemical processes (e.g., hydrolyzes) had become a contributing factor over time, or both.

The total amount of HMW DNA averaged per treatment group was <16% (Table 2), raising the question as to what caused the initial stages of degradation. The physical handling of DNA during the extraction and pipetting processes can cause a mechanical break to double‐stranded DNA. A protocol that limits the movement and manipulation of DNA would likely result in higher proportions or longer fragments (Mayjonade et al., 2016). This could be preferred a method when DNA is extracted for the sequencing of a reference genome or long‐read sequencing for detecting structural variation. A subset of the DNA would likely also have degraded within the first 24 hr after sampling. DNA was relatively stable for the first month when stored in DESS (Table 2), suggesting that degradation in the first 24 hr was limited. For the purposes of this study, the effects experienced in the initial 24 hr were not a specific interest, as the key goal was to evaluate how fast DNA degrades when molecular facilities are not at hand.

For this study, we evaluated the performance of two commonly used preservation solutions, which offer an economical solution for sample preservation. Solutions such as RNAlater (Invitrogen^©^) and DNA/RNA shield (Zymo research^©^) provide additional options for sample preservation but come at an increased cost. These solutions are of increasing interest as they are capable of preserving both DNA and RNA. RNAlater works similar to DESS, where metal chelation by EDTA inactivates DNA degrading enzymes such as DNase (Malmstrom, 2015). An important difference is that DMSO alters methylation profiles, rendering samples stored in DESS unfit for epigenetic research (Kasai & Kawai, 2009). The absence of DMSO in RNAlater and DNA/RNA shield potentially limits the transport of other components through cell membranes, which could be a problem for tough tissue samples (Notman, Noro, O'Malley, & Anwar, 2006). Another interesting feature of both RNAlater and DNA/RNA shield is that it has been designed to preserve DNA under ambient conditions (Gorokhova, 2005). Also, lyophilization (freeze drying) is a commonly applied method for the preservation of biological material. Although DNA is preserved well using lyophilization, this method is far less cost‐effective and requires a specialist piece of equipment which is unpractical in the field (Anchordoquy & Molina, 2007). Finally, biostability molecules and dry‐state DNA stabilization systems (e.g., Biomatrica^®^ DNAstable^®^ or polyvinyl alcohol, PVA) provide alternatives to the widely applied TE buffer for long‐term storage of purified DNA (Clement, Whitney, Muller‐Cohn, & Muller, 2012; Ivanova & Kuzmina, 2013). These compounds have been found to preserve purified DNA better at nonfreezing temperatures, which can be particularly useful when shipping DNA over long distances to sequencing facilities.

## 5 CONCLUSIONS

The application of NGS will continue to increase over the coming decade, and an increasing number of studies will be conducted on nonmodel species sampled in the field. Ongoing reductions in sequencing costs and the large selection of services offered by sequencing providers (from DNA extraction to genome annotation) are making genomic research accessible to a large scientific audience. Sample collection and preservation are the first and crucial step that will allow us to gain novel insights regarding animal ecology, demography, and evolution using genomic methods. Our study highlights the need for careful evaluation of sample preservation and provides key considerations for anyone planning sampling DNA for genomic research.

## CONFLICT OF INTEREST

None declared.

## AUTHOR CONTRIBUTION

**Tom Oosting:** Conceptualization (lead); Data curation (lead); Formal analysis (lead); Methodology (lead); Visualization (lead); Writing‐original draft (lead); Writing‐review & editing (equal). **Elena Hilario:** Formal analysis (supporting); Methodology (supporting); Resources (supporting); Software (supporting); Supervision (supporting); Writing‐review & editing (supporting). **Maren Wellenreuther:** Conceptualization (supporting); Methodology (supporting); Resources (supporting); Supervision (equal); Writing‐original draft (supporting); Writing‐review & editing (equal). **Peter A. Ritchie:** Conceptualization (supporting); Methodology (supporting); Resources (equal); Supervision (equal); Writing‐original draft (supporting); Writing‐review & editing (equal).

## Supporting information

Appendix S1

Appendix S2

## Data Availability

All data files, python, and R scripts are available as Appendices S1 and S2 and uploaded to Dryad: https://doi.org/10.5061/dryad.b5mkkwh9k.
