# Supplementary material for: DNA degradation in fish: Practical solutions and guidelines to improve DNA preservation for genomic research
Source: Ecol Evol. 2020 Jul 13;10(16):8643–51. doi: 10.1002/ece3.6558 (PMC7452763; doi:10.1002/ece3.6558)
Supplement: Supplementary file 1 — Appendix S1 [file ECE3-10-8643-s001.docx]

**Supplementary information**

**DESS (DMSO/ EDTA/ NACl) protocol**

**Disclaimer*
This protocol was adopted from Melissa Yoder ([melissay@ucr.edu](mailto:melissay@ucr.edu)). The original document can be found at: <http://www.faculty.ucr.edu/~pdeley/lab/melissa/DESS_protocol_f.doc>

Recipe:
0.25M disodium EDTA pH 8.0
20% DMSO (Dimethyl Sulfoxide)
NaCl saturated

**The most difficult and time-consuming part of making DESS solution is waiting for the EDTA to dissolve, therefore make it easier by preparing a large volume of EDTA at double the molarity (0.5M EDTA) that can used at a moment’s notice.

Below are the directions for making large volumes of DESS using a stock solution of 0.5M EDTA. Also directions on how to make small volumes of DESS the original, more difficult way using 0.25M EDTA. Both directions can be easily adapted to make either small or large volumes of EDTA. NOTE: Be patient when making any EDTA solution because it will not even begin to dissolve until it reaches a pH of ~7.

**See the FAQ section below for answers about the formation of crystals at the bottom of the solution. If you still have questions, email me (Melissa Yoder) at melissay@ucr.edu and I will do my best to answer your questions.

New easier way to make DESS:
First make a stock solution of 0.5M EDTA. If the stock solution is already present then proceed to the section below that says ‘2L DESS.’

2L 0.5M disodium EDTA
Disodium EDTA FW 374.24 372.24g
5M NaOH To pH EDTA to 8.0
Deionized water Bring final volume to 2L

1. Measure out 372.24g of disodium EDTA, add 500mL deionized water to the EDTA, and enough 5M NaOH to pH the solution to 8.0 (this can be as much as 500mL in some cases). The EDTA will start to dissolve around a pH of 7.0, which may take several hours, so be patient.

**Make sure to use disodium EDTA salt, otherwise more NaOH is needed to pH the EDTA.

2. Bring the final volume to 2L with deionized water.

2L DESS:
0.5M disodium EDTA 1L
Dimethyl Sulfoxide (DMSO) 400mL
Deionized water 600mL
NaCl Enough to saturate the solution ~300g

1. Measure out and mix the first three chemicals.
2. Add enough NaCl to saturate the solution and dissolve. This means using roughly 300–400g but this may vary depending on several factors including ambient temperature, etc. Therefore it is best to add 300 grams and, if everything dissolves then add more salt until it seems like it just isn’t dissolving any more. This may take several minutes to hours, so be patient.

## **Rapid Salt-extraction protocol** (adapted from Salah & Martinez 1997)

Ingredients:

- 20 mg / mL proteinase K
- Extraction buffer (0.4 M NaCl, 10 mM Tris-HCl pH 8.0, and 2 mM EDTA pH 8.0 10% sodium dodecyl sulfate (warm to re-dissolve)
- TE buffer (10 mM Tris, 1 mM EDTA, pH 8.0)
- 5M sodium chloride (NaCl saturated dH_2_O: Autoclave)
- 100% isopropanol chilled
- 70% ethanol chilled
- Distilled water in squirt bottle

Equipment:

- Incubator (rotating)
- Refrigerated centrifuge
- Pipettes
- 1.5 mL microcentrifuge tubes
- Forceps
- Scissors
- Kimwipes® (fine tissues)
- Bunsen burner

**Sample preparation**

1. Select the samples to be used from the freezer and hold on ice during sample prep.
2. Remove the fin-clip/tissue from ethanol storage and cut a portion onto a clean Kimwipe in a Petri dish/glass slide.
3. **Weigh ~20–40 mg of sample into a 1.5 mL microcentrifuge tube** and return fin clip/tissue to storage. Squash out the DESS/ethanol and weigh. For tissue, consider rinsing in water, and re-drying with a Kimwipe.
4. Clean forceps and scissors before further sampling:
   1. Wash with distilled water
   2. Wipe with clean paper towel
   3. Wash with ethanol
   4. Sterilise by passing through a flame

**Cell lysis**

1. Add **480 µL cell lyses buffer or** Heat tubes to 80°C for 5 min to inactivate enzymes such as DNase.
2. Cool samples on ice.
3. Add **5 µL of 20 mg/mL proteinase K** to each tube.
4. Incubate at 56°C for 1.5–3 hr. Invert the sample every 15 minutes during digestion to mix or set orbital mixer to 300rpm throughout digestion (all samples were continuously rotated during incubation). If necessary, add more proteinase K. (*Overnight option*: incubate at 37°C overnight – all samples in this study were not incubated overnight)

**Protein precipitation and removal**

1. Spin max speed 5 min.
2. Transfer supernatant to new tube using wide bore tips. Keep pipette tip close to the surface and occasionally mix surface layer with pipette tip without dislodging pellet in the bottom of the tube.
3. **Add 320 µL of 5M sodium chloride** to each tube and mix by inverting the tubes 60 times.
4. Spin max speed 5 min.
5. Transfer supernatant to new tube using wide bore tips.

**DNA precipitation**

1. **Add 525µL of chilled 100% isopropanol** to the supernatant, inverted several times, and hold at -20°C for ~1 hr- overnight if more convenient.
2. Centrifuge tubes for 20 min at 13K xg and 4°C.
3. Carefully remove supernatant without disturbing pellet.
4. **Add 1mL of chilled 70% ethanol** to tubes and invert several times.
5. Centrifuge for 10 min at 13K xg and 4°C.
6. Remove the supernatant and air dry the pellet for approximately 15 min at 25°C. Do not over-dry your samples.

**DNA rehydration**

1. **Add 100 µL of TE buffer** to the dried pellet and leave to rehydrate for >30 min (or overnight)
2. Gently agitate (flick) tube to re-suspend DNA.
3. Store DNA in fridge until checking quality (no longer than 48 hrs) or store in freezer for long-term storage. Remember to label your samples well and date them.
